# Disparities between HIV patient subgroups in Oman: An analysis of the 2019 cascade of care

**DOI:** 10.1371/journal.pone.0254474

**Published:** 2021-07-09

**Authors:** A. Elgalib, S. Shah, A. Al-Wahaibi, Z. Al-Habsi, M. Al-Fouri, R. Lau, H. Al-Kindi, B. Al-Rawahi, S. Al-Abri

**Affiliations:** Directorate General for Disease Surveillance and Control, Ministry of Health, Muscat, Oman; University of Ghana College of Health Sciences, GHANA

## Abstract

**Background:**

The HIV cascade of care is a framework for monitoring HIV care, identifying gaps and informing appropriate interventions. This study aimed to describe the cascade of care in Oman in 2019 and highlight disparities at the sub-population level.

**Methods:**

We used the UNAIDS Spectrum modelling software to estimate the number of people living with HIV. A national HIV surveillance database was used to identify Omani people (≥13 years old) diagnosed with HIV from 1984 through December 2019. We calculated the cascade indicators as of 31 December 2019 stratified by sex, age, HIV risk factor, residence, and region of HIV care. We also performed multivariate logistic regression to determine the predictors of attrition at linkage, retention, on ART, and viral suppression.

**Results:**

As of December 2019, the estimated number of people living with HIV in Oman was 2440. Out of the estimated number of people living with HIV, 69% were diagnosed, 66% were linked to care, 61% were retained in care, 60% were on ART, and 55% were virally suppressed. Of the 1673 diagnosed individuals, 96% were linked to care, 88% were retained in care, 87% were on ART, and 81% were virally suppressed. People who received HIV care outside Muscat had the largest attrition (11% loss) in the transition from linkage (97%) to retention (86%). Similarly, people aged 13–24 years had the largest attrition (13% loss) from “on ART” (88%) to viral suppression (75%). Logistic regression showed that both not reporting a specific HIV risk factor and receipt of HIV care outside Muscat independently predicted attrition at each cascade stage from linkage to care through viral suppression.

**Conclusions:**

Our findings identified substantial disparities across various subpopulations along the cascade of care in Oman. This analysis will be invaluable in informing future interventions targeting patient subgroups who are at the highest risk of attrition.

## Introduction

HIV infection remains a significant global public health threat, despite the substantial gains in the fight against the HIV epidemic. The Joint United Nations Programmes on HIV/AIDS (UNAIDS) recently published report showed that the estimated number of people living with HIV at the end of 2019 was 38 million (1). New HIV infections and AIDS-related deaths in 2019 were estimated at 1.7 million and 690, 000, respectively. As of December 2019, 81% of the estimated people living with HIV knew their HIV status, 67% were on antiretroviral therapy (ART), and 59% were virally suppressed. The UNAIDS report indicated that all global targets for 2020 would be missed and called for addressing the entrenched inequalities that continue to drive the HIV epidemic [1].

The HIV cascade of care (referred to as the cascade hereafter) is a framework for monitoring HIV care; the importance of its role in identifying gaps and informing appropriate interventions is increasingly recognised [2]. The components of the HIV cascade are the diagnosis, linkage to care, retention in care, ART uptake, and viral suppression [3]. Attrition across this multi-step process among various patient subgroups is not homogeneous [4–7]. Hence, stratifying the cascade by various sociodemographic and clinical characteristics can detect heterogeneity at the sub-population level and allow a “granular” approach that focuses on vulnerable populations with the greatest need.

Our group has recently reported the 2018 cascade of HIV care in Oman. The estimated number of people living with HIV in Oman was 3030; 1532 (50.6%) of whom were diagnosed [8]. The proportion of people who were linked to care, retained in care, on ART, and with viral suppression (<1,000 copies/mL) out of those who were diagnosed (n = 1532) were 96.3%, 89.5%, 98.2%, and 87.5%, respectively. Out of the estimated and diagnosed people living with HIV, 37.3% and 73.7%, respectively, were virally suppressed [8]. Identifying gaps across the HIV care continuum and patient subgroups at higher risk for poor clinical outcomes is crucial to inform targeted interventions and progress towards ending the AIDS epidemic in Oman by 2030. Hence, we used a national HIV surveillance dataset to describe the cascade in Oman in 2019 and highlight disparities in health outcomes by stratifying cascade steps by sex, age, HIV risk factor, residence, and region of HIV care.

## Methods

### Setting

The Sultanate of Oman is located in the Arabian Peninsula, with a total population of 4,601,706 of whom 2,022,470 (44%) are non-Omanis [9]. The HIV incidence and prevalence are low in Oman [10, 11]; the UNAIDS data show that HIV incidence (0.07 per 1000 adults) and prevalence (0.2 per 1000 adults) in Oman were stable in 2010–2019 [11]. The Omani National AIDS Programme (NAP) was formed in 1996; it is now part of the Directorate General for Disease Surveillance and Control (DGDSC) at the Ministry of Health. In December 2015, national guidelines recommended ART for all irrespective of CD4 cell count [12]. Under the Universal Health Coverage, HIV care and ART are provided free of charge to all people living with HIV in Oman. There are currently 14 public treatment centres in the country; 4 are in Muscat (all are consultant-led), and ten are outside Muscat (one is consultant-led). All treatment centres have access to CD4 testing, with a national central public health laboratory (CPHL) located in Muscat that is responsible for performing HIV viral load (VL) and genotypic resistance testing.

### Data source

We used a national HIV surveillance database. The Ministry of Health has been collecting individual-based HIV data since 1984. Public health law for communicable diseases in Oman mandates notifying HIV infection within seven days of diagnosis. Data on notification forms include demographics, HIV risk factors, the reason for HIV testing, baseline CD4 cell count, baseline HIV VL, and ART details. The CPHL shares the HIV VL testing data with the NAP on a bimonthly basis. Staff at the NAP regularly collect follow-up clinical data from all HIV clinics.

### Population

Omani people (≥13 years old) diagnosed with HIV from 1984 through December 2019. We excluded patients who were known to have died (n = 28) or who had emigrated (n = 7). Non-Omani patients were also excluded.

### Definitions of the cascade steps

- HIV diagnosis: positive HIV serology or point-of-care test, which was confirmed by a positive Western blot or nucleic acid-based test.
- Linkage to care: receiving HIV-related services (CD4 cell count or HIV VL test) after HIV diagnosis.
- Retention in care: having at least one clinical care episode with documented HIV VL measurement in 2019.
- On ART: having evidence of ART prescription and dispensing in 2019.
- Viral suppression: achieving a plasma HIV VL <1,000 copies/mL at the most recent measurement in 2019.

### Variables

The key variables used for univariate (descriptive) analysis were sex, age as of 31 December 2019 (13–24, 25–49 and ≥50 years), risk factor (heterosexual, men who have sex with men (MSM), other and unknown), residence (Muscat and outside Muscat) and region of HIV care (Muscat and outside Muscat). “Other” HIV risk category included people who inject drugs (PWID), vertical transmission, and blood transfusion. The risk factor was categorised as “unknown” when patients did not report any specific risk factor for HIV infection. Other variables included marital status, the reason for HIV testing, year of HIV diagnosis, hepatitis B and C status, baseline CD4 cell count, baseline HIV VL, most recent HIV VL, ART details, and time on ART (years).

### Study design and statistical analysis

We performed a cross-sectional analysis to calculate the cascade indicators as of 31 December 2019. We conducted a descriptive univariate analysis for sociodemographic and clinical parameters for people diagnosed with HIV; categorical variables were reported as frequency and percentage, and continuous variables were reported as medians and interquartile ranges. We used the UNAIDS Spectrum modelling software [13] to estimate the number of people living with HIV. We calculated the prevalence-based (estimated) cascade indicators by dividing the number of people at each step of the cascade by the estimated number of persons living with HIV [14]. We also measured the diagnosed-based cascade indicators, stratified by sex, age, risk group, residence, and region of HIV care, using the number of diagnosed individuals as a denominator for each stage of the cascade [14].

We performed multivariate logistic regression to determine the predictors of attrition at linkage, retention, on ART, and viral suppression. We adjusted models for the following variables: sex, age, HIV risk group, residence, and region of HIV care. We included all predictor variables in the multivariate model irrespective of their statistical significance in the univariate analysis. The adjusted odds ratio (aOR) was reported at a 95% confidence interval (CI). We analysed data using R Software.

#### Ethical considerations

We used programme data collected for routine patient care. Permission to use the data was obtained from the DGDSC, at the Ministry of Health, who deemed the study as a public health programme evaluation. Hence, institutional review board approval and informed consent were not sought. There was no interaction with human subjects, and the conduct of the study did not expose patients to any risk. The data were not accessed by any other third party other than the study team. We strictly maintained anonymity and confidentiality throughout the study by using non-personal identifiers such as the patient’s index number in the NAP registry.

## Results

### Sociodemographic and clinical characteristics

As of 31 December 2019, a total of 1673 people were living with HIV in Oman. The majority were males (69%), with a median age of 39 years (interquartile range [IQR], 31–48 years). Patients aged 25–49 years accounted for 73% of the total cohort; only 6% aged 13–24 years (Table 1). Most individuals (64%) self-identified as heterosexual, and 10% did not disclose their HIV risk behaviour. Approximately a third of people lived in Muscat (32%), and 60% were receiving HIV care in Muscat.

**Table 1:** Sociodemographic and clinical characteristics of adult and adolescent Omani HIV patients who were diagnosed with HIV by December 2019 (N = 1673)*.

Characteristic	n (%) *
Sex	
Male	1149 (69%)
Female	524 (31%)
Age, years, median (IQR)	39 (31–48)
Age group (years)	
13–24	99 (6%)
25–49	1215 (73%)
≥ 50	359 (21%)
HIV Risk factor	
Heterosexual	1079 (64%)
MSM	314 (19%)
Other	113 (7%)
Unknown	167 (10%)
Reason for HIV test (n = 1151)	
HIV-related symptoms	376 (33%)
HIV contact	197 (17%)
Antenatal screening	117 (10%)
Patient request	103 (9%)
Other	358 (31%)
Year of HIV diagnosis	
Pre 2011	660 (40%)
2011–2015	474 (28%)
2016–2019	539 (32%)
Baseline CD4 count, cells/mm^3^, median (IQR)	246 (104–411)
Baseline HIV viral load, copies/mL, median (IQR)	49,618 (5,060–197,177)
HBsAg (n = 1362)	
Positive	88 (6%)
Negative	1274 (94%)
HCV antibodies (n = 1349)	
Positive	73 (5%)
Negative	1276 (95%)
ART (ever during study)	
Yes	1552 (93%)
No	121 (7%)
Time on ART (years) (n = 1552)	
<1	161 (10%)
1–4	444 (29%)
>4	947 (61%)
ART details (n = 1552)	
NNRTI-based	983 (63%)
PI-based	459 (30%)
INSTI-based	50 (3%)
Others	60 (4%)
Region of residence	
Muscat	535 (32%)
Outside Muscat	1138 (68%)
Region of HIV care (n = 1637)	
Muscat	976 (60%)
Outside Muscat	661 (40%)
Marital status (n = 1581)	
Married	861 (54%)
Divorced/widowed	127 (8%)
Single	593 (38%)

* Unless otherwise stated.

ART, antiretroviral therapy; PWID, people who inject drugs; INSTIs, integrase inhibitors; IQR, interquartile range; HCV, hepatitis C Virus; HIV, human immunodeficiency virus; HBsAg, hepatitis B surface antigen; MSM, men who have sex with men; NRTIs, nucleoside reverse transcriptase inhibitor; NNRTI, non-nucleoside reverse transcriptase inhibitor; PI, protease inhibitor.

### Prevalence- based cascade estimates

Based on Spectrum modelling for Oman, there are an estimated 2440 people living with HIV, 69% (95% CI: 57–90) were diagnosed. Out of the estimated number of people living with HIV, 66% (95% CI: 50.49–79.73) were linked to care, 61% (95% CI: 46.47–73.38) were retained in care, 60% (45.94–72.53) were on ART, and 55% (95% CI: 42.5–67.1) were virally suppressed. The proportion of women who knew their HIV infection was 71% (524/740) compared with 68% (1149/1700) in men; *p* value 0.115.

### Diagnosed- based cascade estimates

**Fig 1:**
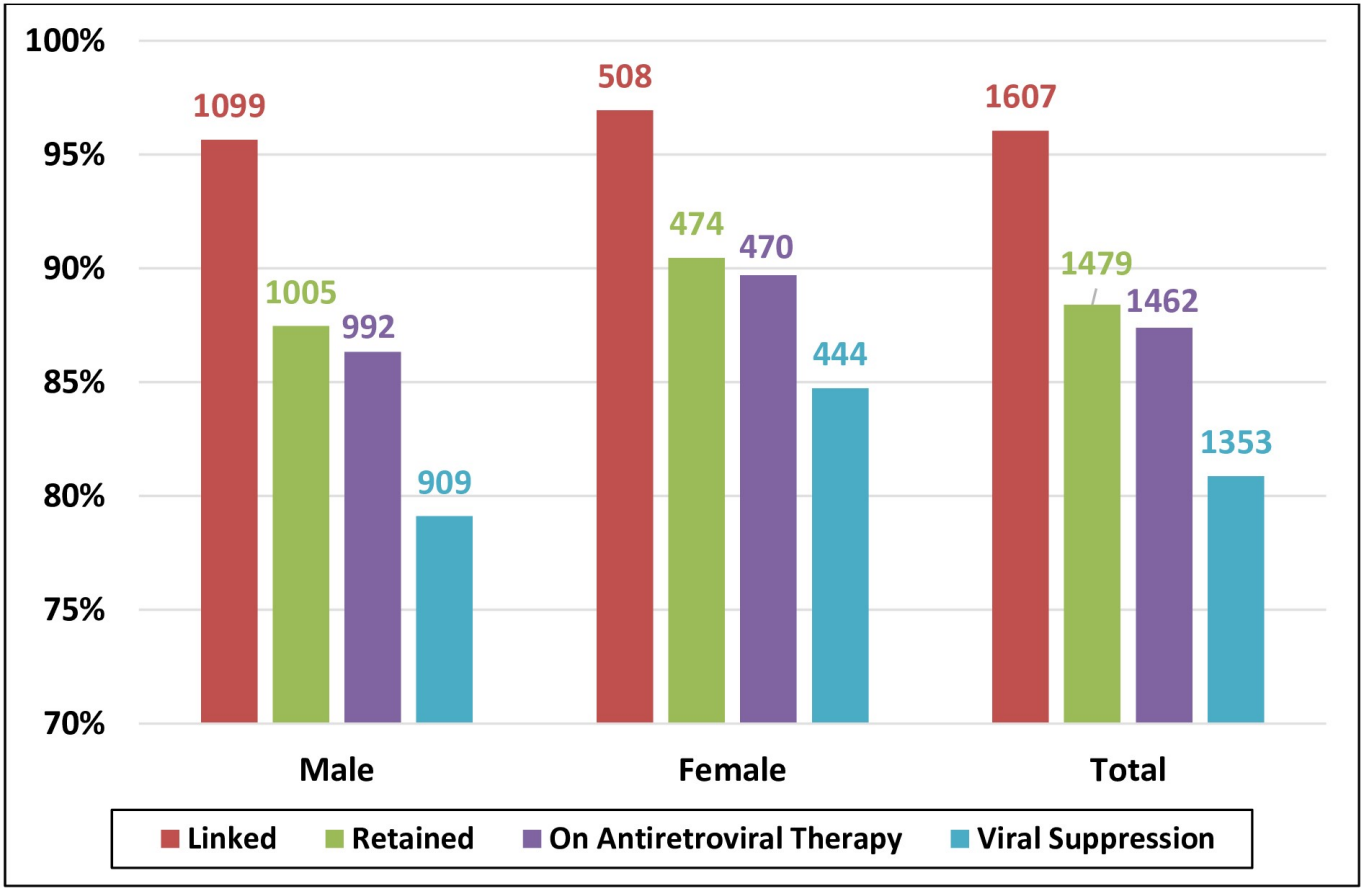
Diagnosed-based 2019 HIV care cascade steps stratified by sex; out of diagnosed people living with HIV in Oman (N = 1673).

Of the 1673 diagnosed individuals, 96% were linked to care, 88% were retained in care, 87% were on ART, and 81% were virally suppressed (Fig 1). Out of the 1462 people who were on ART, 93% achieved viral suppression. Stratification by sex showed that men had worse outcomes than women at each cascade point (Fig 1). The percentage of women who were on ART was 90% compared with 86% in men; *p* value 0.055. The percentage of women with viral suppression was 85% compared with 79% in men; *p* value 0.007.

**Fig 2:**
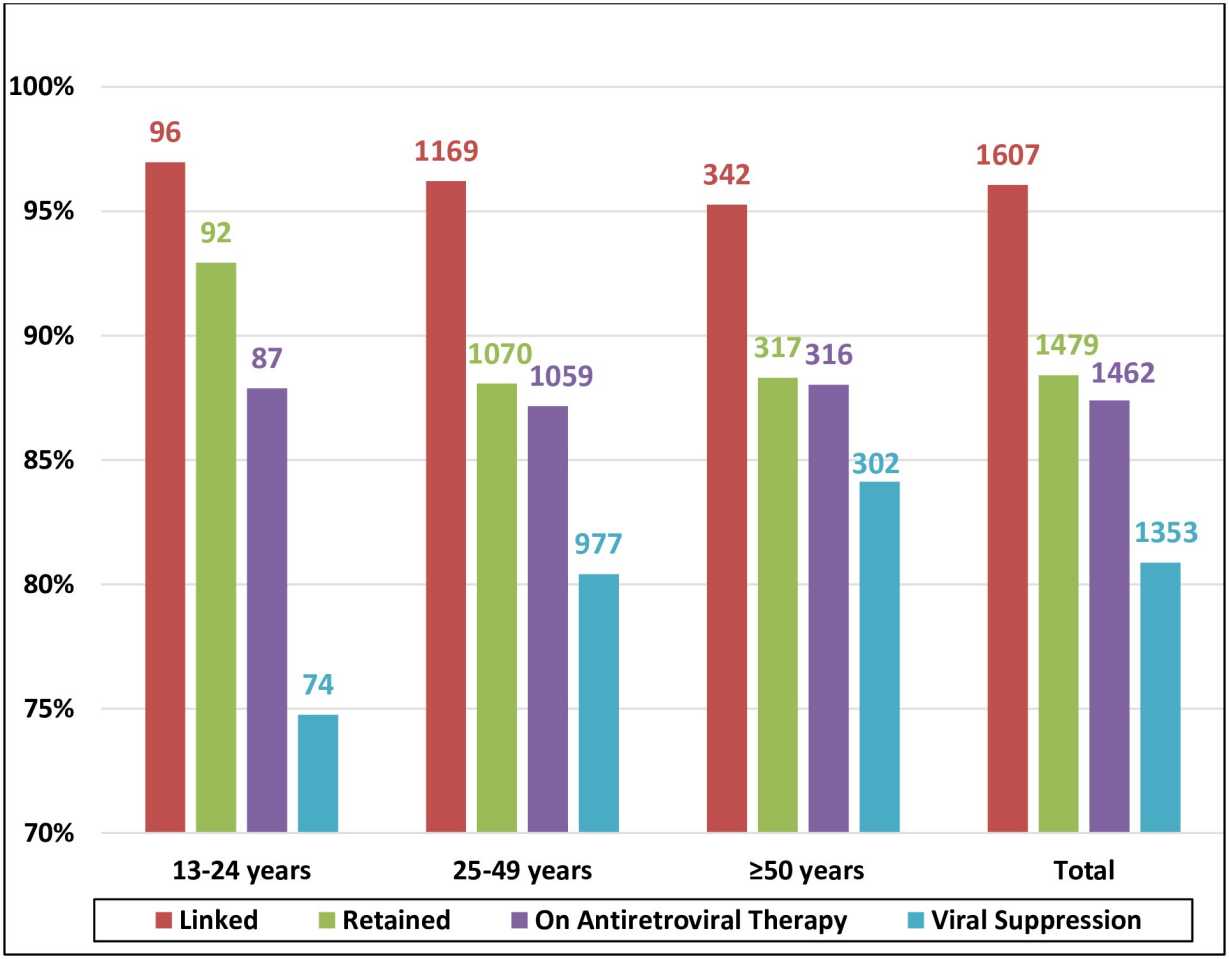
Diagnosed-based 2019 HIV care cascade steps stratified by age group; out of diagnosed people living with HIV in Oman (N = 1673).

**Fig 3:**
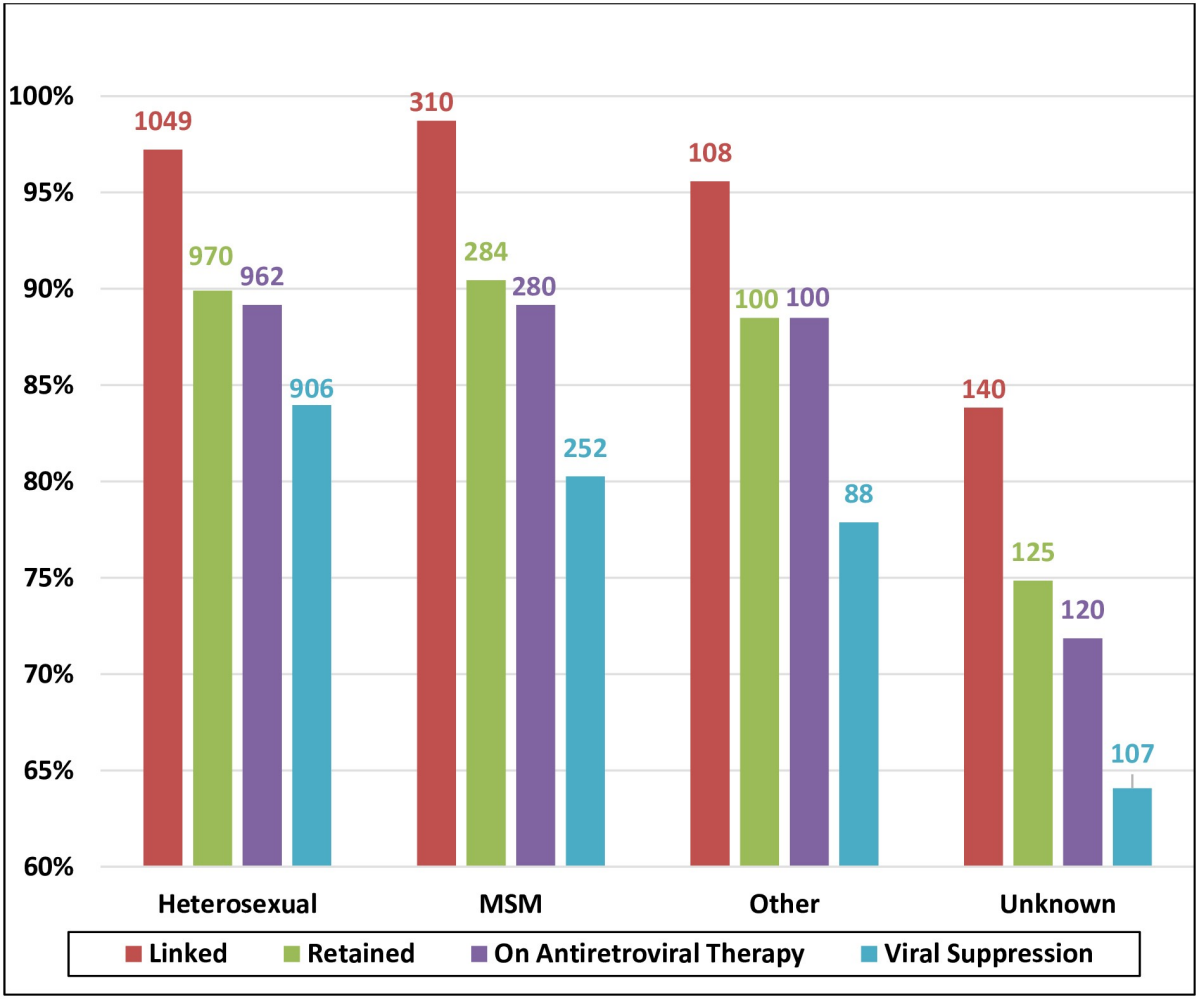
Diagnosed-based 2019 HIV care cascade steps stratified by HIV risk category; out of diagnosed people living with HIV in Oman (N = 1673). MSM = men who have sex with men.

Fig 2 illustrates the cascade stratified by age. A larger proportion of patients aged 13–24 years were retained in care (93%) compared with patients aged 24–49 years (88%) and ≥ 50 years (88%); *p* value 0.347. By contrast, the rate of viral suppression increased with age. The percentages of persons aged 13–24, 25–49, and ≥ 50 years with viral suppression were 75%, 80%, and 84%, respectively. Fig 3 depicts the cascade of care stratified by HIV risk group. Patients who did not report a specific risk factor for HIV infection had the worst outcomes from linkage to care (84%) through the viral suppression (64%); they also had the largest attrition in the transition from linkage to retention (9% loss).

**Fig 4:**
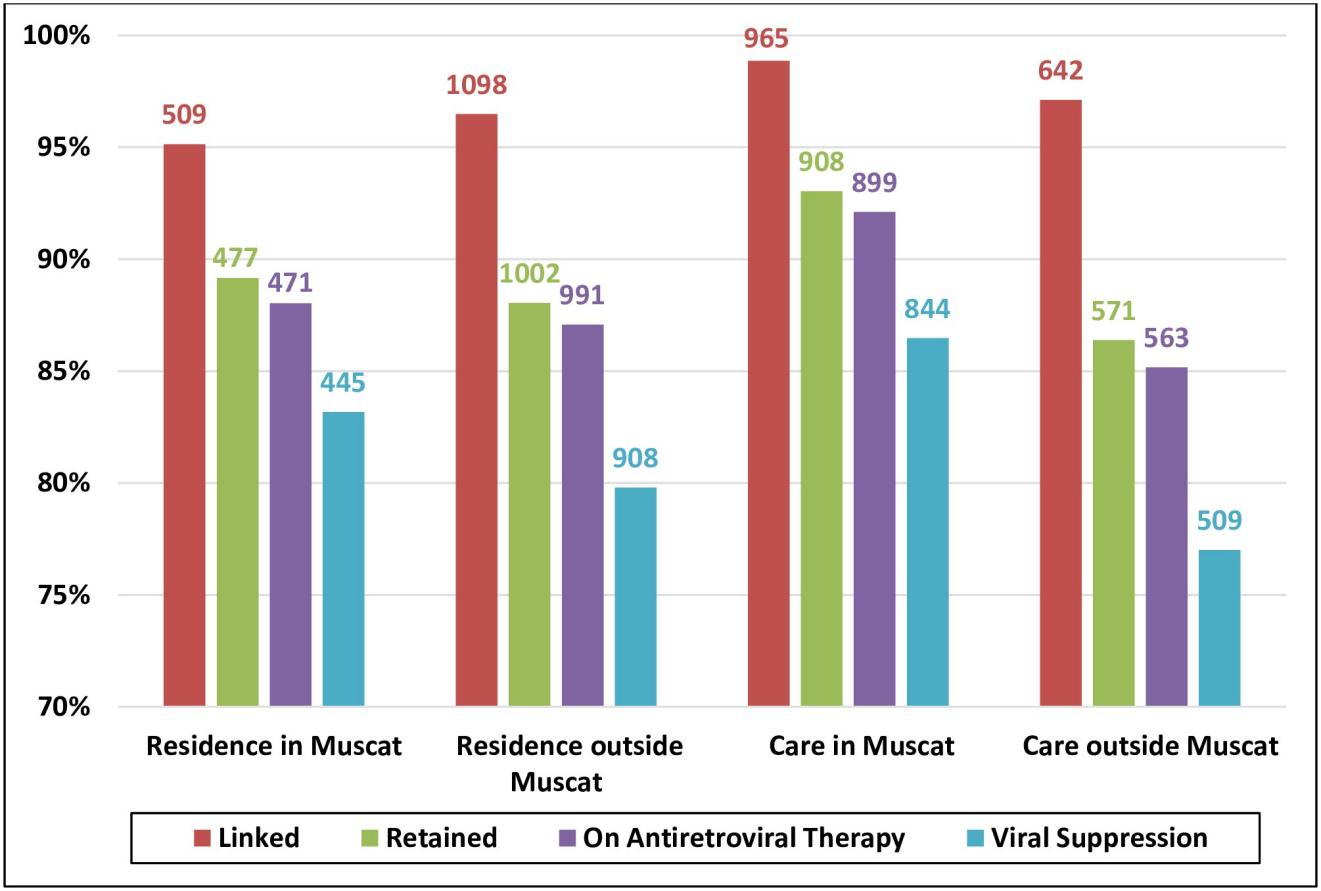
Diagnosed-based 2019 HIV care cascade steps stratified by regions of residence and care; out of diagnosed people living with HIV in Oman (N = 1673).

Compared with patients receiving care in Muscat, those who received care outside Muscat had a lower percentage of viral suppression and larger attrition in the transition from linkage to retention (77% vs. 86%; *p* value <0.001 and 11% vs. 6%; *p* value <0.001, respectively) (Fig 4). Residents of Muscat had similar proportions of retention in care and viral suppression to those who resided outside Muscat (89% vs. 88%; *p* value 0.024 and 83% vs. 80%; *p* value 0.1, respectively).

### Factors associated with attrition across the cascade stages

Table 2 shows the multivariate analysis of factors associated with attrition along the cascade. Sex, age, and region of residence were not significantly associated with attrition at any stage of the cascade. However, younger age had a trend towards significance for attrition at the transition from “on ART” to viral suppression. Compared to patients aged ≥50 years, those aged 25–49 years (aOR 1.38 (95% CI: 0.94–2.03)) and 13–24 years (aOR 1.88 (95% CI: 0.98–3.63)) had higher odds of not achieving viral suppression. Patients who did not report a specific risk factor for HIV infection (compared to heterosexuals) had significantly higher odds for attrition at linkage (aOR 4.05 (95% CI: 1.38–11.93)), retention (aOR 2.04 (95% CI: 1.17–3.57)), on ART (aOR 3.88 (95% CI: 2.01–7.49)) and viral suppression (aOR 2.12 (95% CI: 1.35–3.32)). Similarly, receiving HIV care outside Muscat (compared to receiving HIV care in Muscat) was an independent predictor for attrition at linkage (aOR 2.58 (95% CI: 1.02–6.52)), retention (aOR 2.28 (95% CI: 1.52–3.42)), on ART (aOR 2.17 (95% CI: 1.25–3.78)) and viral suppression (aOR 1.93 (95% CI: 1.41–2.64)).

**Table 2:** Factors associated with attrition at the stages of the 2019 HIV cascade of care for people diagnosed with HIV in Oman as of December 2019 (N = 1673).

Variable	Linkage	Retention	On ART	Viral suppression
	a OR (95% CI)	a OR (95% CI)	a OR (95% CI)	a OR (95% CI)
Sex				
Female	Reference	Reference	Reference	Reference
Male	0.81 (0.29–2.24)	1.14 (0.73–1.77)	1.09 (0.57–2.08)	1.1 (0.77–1.55)
Age category (years)				
13–24	1.51 (0.22–10.44)	0.87 (0.31–2.44)	2.13 (0.66–6.86)	**1.88 (0.98–3.63)**
25–49	1.84 (0.57–5.92)	1.29 (0.81–2.06)	1.64 (0.82–3.29)	**1.38 (0.94–2.03)**
≥ 50	Reference	Reference	Reference	Reference
HIVRisk factor				
Heterosexual	Reference	Reference	Reference	Reference
MSM	0.56 (0.14–2.15)	0.88 (0.54–1.44)	1.13 (0.59–2.16)	1.08 (0.75–1.56)
Other	2.03 (0.39–10.51)	1.0 (0.47–2.16)	0.65 (0.18–2.33)	1.19 (0.67–2.1)
Unknown	**4.05 (1.38–11.93)**	**2.04 (1.17–3.57)**	**3.88 (2.01–7.49)**	**2.12 (1.35–3.32)**
Region of residence				
Muscat	Reference	Reference	Reference	Reference
Outside Muscat	2.11 (0.65–6.83)	1.07 (0.67–1.71)	1.22 (0.64–2.33)	1.17 (0.82–1.68)
Region of HIV care				
Muscat	Reference	Reference	Reference	Reference
Outside Muscat	**2.58 (1.02–6.52)**	**2.28 (1.52–3.42)**	**2.17 (1.25–3.78)**	**1.93 (1.41–2.64)**

aOR, adjusted odds ratio; ART, antiretroviral therapy; CI, confidence interval; MSM, men who have sex with men.

## Discussion

Based on the Spectrum analysis of HIV data for Oman in 2019, 69% of the estimated people living with HIV were aware of their infection, 66% were on ART, and 55% were virally suppressed. Among diagnosed individuals, the overall retention in care (88%) and viral suppression (81%) rates were favourable; however, the former ranged from 75% to 93%, and the latter ranged from 64% to 87% in various stratifications. People who received HIV care outside Muscat had the largest attrition (11% loss) in the transition from linkage to retention. Similarly, people aged 13–24 years had the largest attrition (13% loss) from “on ART” to viral suppression. Both receipt of HIV care outside Muscat and not reporting a specific HIV risk factor independently predicted attrition at each cascade stage.

The proportion of people living with HIV who were aware of their HIV infection in Oman is well below the global average of 81%, yet it exceeds the regional average of 52% [1]. Of note, we are unable to explain the drop in the estimated number of people living with HIV in Oman from 3030 in 2018 [8] to 2440 in 2019; however, this might be related to a change in the methodology used in the UNAIDS Spectrum modelling software [13]. Given the high levels of treatment coverage and viral suppression, we observed in our cohort, reducing the prevalence of undiagnosed HIV remains a major challenge to maximising the individual and public health benefits of ART in Oman. Facility- and community- based HIV testing strategies are urgently needed. Previous studies have shown that routine HIV testing in medical admission units and accident and emergency departments in areas of high HIV prevalence (defined as > 1 in 1000) is feasible, acceptable, and effective [15, 16]. The NAP has data on HIV prevalence at governorate’s level that can guide these initiatives as well as self-testing and other innovative approaches. A recent systematic review indicated that expanding self-testing and outreach projects to key populations with facilitated linkage can increase rates of HIV diagnosis and linkage to HIV services [17].

The suboptimal viral suppression rate among young persons in this study might be attributed to drug resistance and poor adherence to ART, as a result of mental health problems [18–20] HIV-related stigma, and non-disclosure of HIV status to family members, which results in weak social support network [21, 22]. Furthermore, the observed large attrition in the transition from retention in care to “on ART” among the same age group might be explained by the reluctance of physicians to initiate ART in this cohort due to lack of patients’ readiness and willingness to start ART. Delaying ART initiation until patients are ready might reduce the risks of treatment failure and emergence of a drug-resistant virus; however, these benefits should be carefully weighed against the risks of disease progression and HIV transmission in the absence of ART. Adolescent-friendly HIV services that meet the particular care needs for this vulnerable cohort are needed in Oman.

Among all patient subgroups, individuals who did not report a specific risk factor for HIV infection had the worst outcomes at all cascade steps. People who chose not to disclose their HIV risk behaviour could belong to key populations, such as MSM and sex workers, and fear of judgment, privacy breaches, HIV stigma, violence, and criminal prosecution might deter them from revealing their HIV risk [23, 24]. A poor provider-patient relationship might also preclude disclosure of HIV risk behaviour. Reassurance about confidentiality and a non-judgemental approach by well-trained HIV caregivers would enhance trust and openness, with consequent improvement in recognition of specific HIV risks, and targeted interventions aimed at improving health outcomes.

The regional (outside Muscat) HIV treatment centres are staffed by general physicians and not as well-resourced as clinics in Muscat, which are led by consultants with easier access to diagnostics and ancillary services, such as mental health support. These provider-related elements might explain the observed disparities in performance between regional and central clinics; however, patient-related factors, including psychosocial aspects, might have contributed too. Besides, fear of confidentiality breaches and HIV-related stigma and discrimination could hinder engagement with HIV services and adherence to ART in smaller communities [25, 26]. It is vital to maintain the decentralised HIV care in the country to improve access to HIV services. However, strengthening the technical and logistic support to regional clinics is necessary along with further research to understand the drivers of the regional heterogeneity across the cascade.

The main strength of our analysis is the ability to calculate prevalence-based cascade, which has identified a high proportion of undiagnosed HIV in the country. Moreover, we used population-based, with individual-level, data derived from a national HIV surveillance system with input from all 14 HIV treatment centres, and the CPHL in the country. A limitation of this study is the limited comparability of its findings to similar published cascades because of the heterogeneity in data sources and definitions of cascade elements [27]. For instance, we defined retention in care as having at least one clinical care episode with a documented HIV VL measurement in 2019 while some other studies used a definition of two clinical episodes, three months apart, in a calendar year; our definition might have resulted in overestimating the retention of care rate. We also used an HIV VL of <1,000 copies/mL as a cut-off for viral suppression to facilitate monitoring the progress towards UNAIDS targets and the change over time, as we used the same cut-off for the cascades in Oman in 2015–2018 [8]. Nonetheless, this further limited the comparability of our data as some reports used an HIV VL of <200 copies/mL as a cut-off for viral suppression. Another limitation of our analysis, we were not able to stratify the cascade by psychosocial factors, such as income, education, mental health and substance misuse, which have been shown to impact the clinical outcomes [26, 28]. Exclusion of patients who were known to have died or had emigrated might have resulted in overestimating the rate of viral suppression as excluded patients might have failed to achieve viral suppression. However, 50% (14/28) and 88% (7/8) of patients who had died and emigrated had HIV VL <1000 copies/ml at or closest to the time of death and emigration, respectively. Finally, criminalisation and social disapproval of high-risk behaviours in Oman might have resulted in overestimating the proportion of heterosexuals in our cohort; fears of stigma and prosecution may preclude MSM and PWID from disclosing their real HIV risk.

In summary, our data demonstrate substantial disparities in retention in care and viral suppression among various subpopulations and will be invaluable in informing future interventions. Strategies targeting patient subgroups who are at the highest risk of attrition, including young persons, those denying HIV risk behaviours and those receiving HIV care outside Muscat are required.
